# Balloon-Assisted Percutaneous Transhepatic Antegrade Embolization with 2-Octyl Cyanoacrylate for the Treatment of Isolated Gastric Varices with Large Gastrorenal Shunts

**DOI:** 10.1155/2019/2674758

**Published:** 2019-04-03

**Authors:** Guangchuan Wang, Dongxiao Meng, Guangjun Huang, Qingshan Pei, Lianhui Zhao, Yongjun Shi, Mingyan Zhang, Hua Feng, Junyong Zhang, Chunqing Zhang

**Affiliations:** Department of Gastroenterology, Shandong Provincial Hospital Affiliated to Shandong University, China

## Abstract

**Aims:**

To evaluate the safety and effectiveness of percutaneous transhepatic antegrade embolization (PTAE) with 2-octyl cyanoacrylate assisted with balloon occlusion of the left renal vein or gastrorenal shunts (GRSs) for the treatment of isolated gastric varices (IGVs) with large GRSs.

**Methods:**

Thirty patients with IGVs associated with large GRSs who had underwent PTAE assisted with a balloon to block the opening of the GRS in the left renal vein were retrospectively evaluated and followed up. Clinical and laboratory data were collected to evaluate the technical success of the procedure, complications, changes in the liver function using Child-Pugh scores, worsening of the esophageal varices, the rebleeding rate, and survival. Laboratory data obtained before and after PTAE were compared (paired-sample* t-*test).

**Results:**

PTAE was technically successful in all 30 patients. No serious complications were observed except for one nonsymptomatic pulmonary embolism. During a mean follow-up of 30 months, rebleeding was observed in 4/30 (13.3%) patients, worsening of esophageal varices was observed in 4/30 (13.3%) patients, and newly developed or aggravated ascites were observed on CT in 3/30 (10%) patients. Significant improvement was observed in Child-Pugh scores (p=0.009) and the international normalized ratio (INR) (p=0.004) at 3 months after PTAE. The cumulative survival rates at 1, 2, 3, and 5 years were 96.3%, 96.3%, 79.9%, and 79.9%, respectively.

**Conclusion:**

Balloon-assisted PTAE with 2-octyl cyanoacrylate is technically feasible, safe, and effective for the treatment of IGV associated with a large GRS.

## 1. Introduction

Variceal bleeding from gastric varices is a serious complication of portal hypertension, which is associated with high mortality [1, 2]. Isolated gastric varices (IGVs), which are special types of gastric varices located in the gastric fundus, frequently drain into the left renal vein via a gastrorenal shunt (GRS) [3]. A large GRS may lead to a potential risk of cyanoacrylate migration and pulmonary embolism when endoscopic embolization therapy is performed [4, 5]. Therefore, treatment of IGVs associated with large GRSs is challenging.

Although balloon-occluded retrograde transvenous obliteration (BRTO) has been shown in a number of studies to have reliable clinical results in IGV treatment, it has a relatively long procedure time (a few hours to overnight) and is inherently limited by its association with sclerosant-related complications [6–10]. Moreover, the efferent vessels rather than the afferent vessels are obliterated during the BRTO procedure, and this may potentially results in the development of esophageal varices and an increased risk of esophageal variceal bleeding [7, 11–13]. Therefore, although the traditional BRTO technique is usually used in Asia (predominantly Japan), it is not selected as the first-choice therapy in the West (United States and Europe) [14].

Modified percutaneous transhepatic antegrade embolization (PTAE) with 2-octyl cyanoacrylate (2-OCA) was reported to be effective in the prevention and treatment of esophageal and gastric variceal bleeding in our previous studies [15–21]. The main feature of this technical results in the anterograde embolization of the varices with cyanoacrylate from the transhepatic catheter, which can permanently obliterate the varices and all feeding veins. To overcome the drawbacks of BRTO described above, we introduced percutaneous transhepatic antegrade gastric variceal embolization with cyanoacrylate to treat IGVs with GRSs. Based on BRTO, a balloon was placed at the opening of a GRS in the left renal vein to temporarily occlude and reduce the risk of ectopic embolism from the GRS. This technique is named balloon-assisted PTAE. The aim of this study is to evaluate the technical safety, clinical safety, and effectiveness of this promising approach.

## 2. Methods

### 2.1. Patients

Between May 2010 and August 2015, 30 patients with IGVs associated with GRSs that were treated with PTAE were retrospectively evaluated. All patients had a history of gastrointestinal (GI) bleeding within 3 months. Prior to the procedure, gastric endoscopy was performed to evaluate the severity of gastric varices, and contrast computed tomography and venography (CTV) of the portal vein system was utilized to evaluate the feeding veins and drainage veins of the gastric varices.

The inclusion criteria were as follows: (1) diagnosis of liver cirrhosis by biopsy or clinical examination and imaging, including ultrasound, computed tomography (CT), or magnetic resonance imaging; (2) patients suffered from bleeding within 3 months before being admitted or acute bleeding that achieved hemostasis by pharmacological treatment; (3) IGVs diagnosed by endoscopy with no other potential source of bleeding; (4) a large GRS >5 mm associated with IGVs observed by preoperative imaging, and (5) patients aged between 20 and 75 years old.

Exclusion criteria were as follows: (1) hepatocellular carcinoma or other malignancies, (2) a history of transjugular intrahepatic portosystemic shunt (TIPS) surgery or endoscopic therapy for esophagogastric variceal bleeding, (3) portal vein thrombosis, (4) uncontrolled infection, and (5) ascites (>moderate).

The characteristics of the 30 patients are shown in Table 1. This study included 16 men and 14 women with a mean age of 49.5 years old. Table 1 shows the underlying cause of portal hypertension and the modified Child's classification of the patients.

The form of fundal varices was massively tumorous varices (F3) in 19 (63%) patients and nodular-shaped varices (F2) in 11 (37%) patients. Red spots were observed in 14 patients, and uncontrolled hepatic encephalopathy was observed in 2 patients.

All patients provided informed consent prior to the treatment. This study was conducted retrospectively in accordance with the ethics guidelines of the Declaration of Helsinki and the International Conference on Harmonization Guidelines for Good Clinical Practice.

### 2.2. Treatment Procedures

All patients had IGVs with large GRSs. To ensure that the occlusion of the varices was extensive and thorough, percutaneous transhepatic portal vein catheterization was performed, and 2-octyl cyanoacrylate was then injected via the feeding veins into the entire varices to achieve the obliteration of the varices and their feeding veins. The PTAE procedure was performed with the assistance of a balloon to block the GRS blood flow in each different method. The details of the procedures are described as follows.

#### 2.2.1. Percutaneous Transhepatic Portography

The PTAE procedure was performed under radiological and sonographic guidance as previously described [15–21]. Before the PTAE procedure, ultrasonography was performed in all patients to determine the best access route to enter the portal venous system. Percutaneous transhepatic puncture of the intrahepatic branch of the portal vein was performed using a 22-gauge Chiba needle under sonographic guidance after local anesthesia, and a 5-French sheath catheter was subsequently introduced into the portal vein. A 5-F Cobra or Pig tail catheter (Cordis, USA) was inserted into the splenic vein, and direct portography was then performed to evaluate the index of varices as well as the feeding vessels and draining veins of the gastric varices.

#### 2.2.2. Balloon Occlusion of Blood Flow in GRS

**Figure 1:**
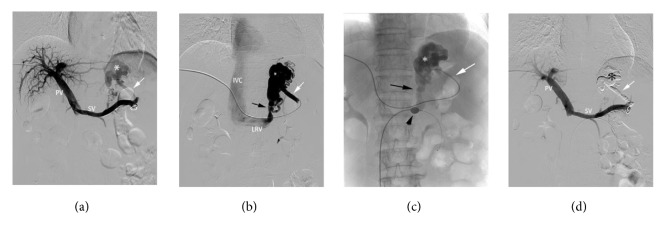
A 56-year-old woman suffering from recurrent upper gastrointestinal bleeding from gastric varices. (a) Direct portography showed gastric varices (white asterisk) from the short gastric vein (white arrow). (b) Angiography of the short gastric vein (white arrow) showed gastric varices (white asterisk) and GRS (black arrow) that drained into left renal vein. (c) A balloon catheter was inserted into the left renal vein (short black arrow) to decrease the blood flow of the GRS (black arrow), and cyanoacrylate was then injected into the varices through the catheter into the feeding vein (white arrow). (d) Direct portography performed immediately after embolotherapy showing the varices (black asterisk) and the feeding vessels (white arrow) were filled with cyanoacrylate. PV, portal vein; SV, splenic vein; LRV, left renal vein; IVC, inferior vena cava.

**Figure 2:**
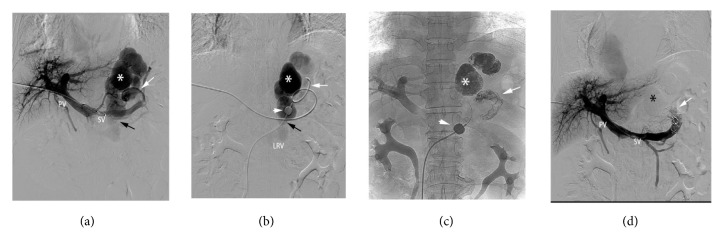
A 65-year-old man suffering from gastric variceal bleeding and encephalopathy. (a) Direct portography showed gastric varices (white asterisk) from the short gastric vein (white arrow) with drainage into the left renal vein through a large GRS (black arrow). (b) A balloon catheter (short white arrow) was introduced into the GRS (black arrow) through the femoral vein, and cyanoacrylate was directly infused into the gastric varices (white asterisk) from the catheter (white arrow) into the posterior gastric vein. (c) Before removal, the balloon catheter (short white arrow) was inflated in place for 1 min until the gastric varices (white asterisk) and the feeding vessels (white arrow) were completely obliterated by the cyanoacrylate. (d) Direct portography performed immediately after the embolotherapy showed the gastric varices (black asterisk) and the feeding vessels (white arrow) were filled with cyanoacrylate. PV, portal vein; SV, splenic vein; LRV, left renal vein.

A 5-8 French sheath catheter was introduced into the right femoral vein. There are two ways to obstruct the out flow of a GRS. (1) If the catheterization of a GRS was difficult, a 5-F Simon II catheter (Cordis, USA) was inserted into the left renal vein, and a balloon catheter (Edwards Lifesciences LLC, Irvine, CA, USA) was introduced into the left renal vein and inflated to occlude the left renal vein (Figure 1) and thereby slow down the blood flow of the GRS. (2) If the catheterization of the GRS was easy, a balloon occlusive catheter (Edwards Lifesciences LLC, Irvine, CA, USA) was introduced into the GRS and inflated to occlude the GRS (Figure 2).

#### 2.2.3. Percutaneous Transhepatic Vein Embolization

Subsequently, a Cobra catheter was inserted into the major feeding vessels of the gastric varices from the portal vein system, such as the coronary gastric vein, posterior gastric vein, or short gastric vein, and a microcatheter was introduced when needed. Then, the cyanoacrylate was directly injected into the gastric varices and their feeding vessels through the catheter under X-ray guidance.

In this study, 2-OCA mixed with 50% lipiodol was used to obliterate the gastric varices. It was slowly injected into the gastric varices under fluoroscopic guidance. Once the gastric varices were completely obliterated, the catheter was slowly withdrawn. The balloon occlusive catheter was then deflated and removed.

Splenoportography was again performed to assess the obliteration of the varices. If any feeding veins (such as the short or posterior gastric veins) were still present, the procedure was repeated until the gastric varices and feeding veins were completely filled with cyanoacrylate.

Finally, the puncture tract within the liver parenchyma was simultaneously embolized with microcoils (Cook Medical, Bloomington, IN, USA). Low molecular weight heparin (100 IU/kg body weight) was subcutaneously administered 24 h after the procedure and daily for 7 d to prevent portal venous thrombosis.

### 2.3. Evaluation of Procedure Efficacy and Follow-Up

Abdominal contrast enhanced CT and endoscopic examination were performed at 1, 3, 6, and 12 months after the procedure and then every 6 months or whenever clinically necessary.

Aggravation of esophageal varices was defined as red spots on esophageal varices during endoscopy. If necessary, the esophageal varices were treated as soon as possible with endoscopic variceal ligation (EVL).

The laboratory data, including hepatic and renal function tests, were analyzed 1 week and 3 months after the procedure. Changes in Child-Pugh scores between data obtained before and after the procedure were also evaluated.

The follow-up evaluation included an assessment of recurrence and bleeding in gastric varices, the aggravation of esophageal varices, complications, and the rate of survival. In patients with rebleeding, endoscopy was performed to identify the cause of the bleeding. Technical success was defined as successful placement of the balloon in the left renal vein or GRS, successful injection of cyanoacrylate into gastric varices and all the feeding vessels from the catheter, and the complete obliteration of varices on subsequent imaging.

### 2.4. Statistical Analysis

The cumulative survival rate and bleeding rate of gastric varices were calculated using the Kaplan-Meier method. The values of the hepatic function tests obtained before and after the procedure were compared using the paired t-test, and the Child-Pugh classification was analyzed using the Wilcoxon t-test. All statistical tests were performed using SPSS version 18 software (SPSS Inc., Chicago, IL, USA), and P< 0.05 was considered statistically significant.

## 3. Results

### 3.1. Technique Results

The PTAE procedure was successfully completed in all 30 (100%) patients. In these 30 patients, 49 feeding vessels were observed, including 18 gastric coronary veins, 15 posterior gastric veins, and 16 short gastric veins. The average dosage of cyanoacrylate was 6.69 ± 2.92 mL (range, 3-16 mL). Obstruction of GRSs with a balloon was performed in 20 patients, and obstruction of the left renal vein was performed in 10 patients. Portal vein pressure (PVP) significantly increased from 24.3 (8-47.8 mmHg) to 29.1 (10-48.6 mmHg) (P<0.01).

Ectopic embolism was observed in one case, in which a small amount of cyanoacrylate migrated into the pulmonary artery because the balloon that was chosen was not large enough. Fortunately, no symptoms were observed in this patient during the follow-up period. Pulmonary infection and deterioration of liver function were observed in another patient. Liver function gradually improved in this patient after the pulmonary infection was controlled. A transient fever with a body temperature higher than 38°C was observed in 4 patients, and abdominal pain requiring medicinal therapy was observed in 2 patients. No intraoperative hemorrhage, renal vein thrombosis, or hemoglobinuria was observed.

### 3.2. Endoscopy Follow-Up

**Figure 3:**
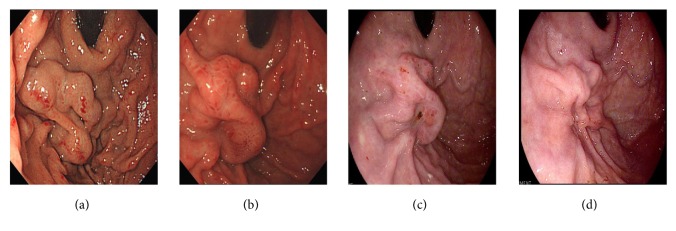
Endoscopic follow-up of the patient shown in Figure 2. (a) Endoscopic image obtained before percutaneous transhepatic antegrade variceal embolization showing large gastric varices. (b) Endoscopic image obtained 1 month after the procedure showing congestion and edema in the mucosa. (c) At 3 months after the procedure, the varices were markedly smaller. (d) One year after the procedure, the gastric varices had mostly disappeared.

During the 30-month follow-up period (2–78 months), all 30 patients were followed up by endoscopy. At 3-4 weeks after the procedure, congestion and edema in the mucosa were relieved, and the mucosa showed chronic inflammation. From 1 to 3 months after injection, the cyanoacrylate glue began to slough off from the submucosa. Cyanoacrylate expulsion was observed in 19 patients (63.3%) within 3 months after PTAE. Of the 25 patients who were followed up for more than 1 year, cyanoacrylate expulsion was completed within 1 year of the procedure in all patients (Figures 3(a)–3(d)). After completion or partial expulsion of the cyanoacrylate, the varices had disappeared or shrunk. One year after the PTAE procedure, the gastric varices had disappeared in 20 patients (80%, 20/25) and shrunk markedly in 4 patients (16%, 4/25). Gastric varices recurrence was found in only 1 patient (4%, 1/25) and observed at 5 months after the PTAE procedure. Additional endoscopic histoacryl injection was performed in this patient, who did not return for follow-up until death, which resulted from massive rebleeding at 35 months after PTAE therapy.

**Figure 4:**
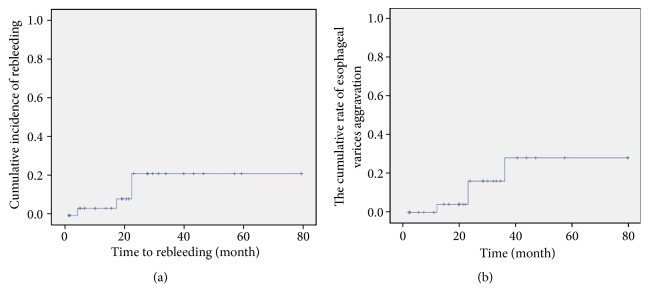
Kaplan-Meier analysis of variceal rebleeding, and aggravation of the esophageal varices. (a) The cumulative rebleeding rate. (b) The cumulative rate of aggravation of esophageal varices.

Aggravation of the esophageal varices under endoscopy was noted in 4 (4/30, 13.3%) patients. The cumulative aggravation rates of the esophageal varices observed at 1, 2, 3, and 5 years were 4.2%, 16.1%, 28.1%, and 28.1%, respectively (Figure 4(a)). Only one patient (1/30) developed active esophageal variceal bleeding (23 months after the PTAE procedure) from esophageal varices aggravation. The esophageal varices were treated with EVL, and no bleeding recurred during the follow-up. Endoscopic variceal ligation was prophylactically performed in the other 3 patients, in whom esophageal varices were observed to be reduced after EVL and no bleeding was observed.

### 3.3. CT Portal Venography Follow-Up

**Figure 5:**
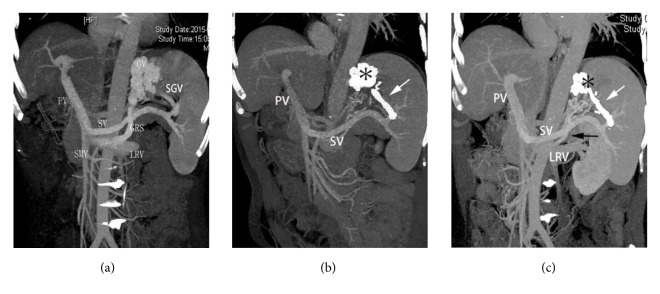
CT follow-up of the patient shown in Figure 2. (a) CT portal venography image obtained before percutaneous transhepatic antegrade variceal embolization showing large gastric varices with drainage by a large GRS. (b) CT portal venography image obtained 3 months after percutaneous transhepatic variceal embolization showing that the gastric varices (asterisk) and their feeding veins (white arrow) were filled with cyanoacrylate. (c) CT portal venography image obtained 1 year after percutaneous transhepatic variceal embolization showing that the cyanoacrylate in the submucosa varices had almost disappeared (asterisk), while the perifundus varices and the feeding veins (white arrow) were still filled with cyanoacrylate, similar to result observed before follow-up. Moreover, the gastrorenal shunt was reserved (black arrow). GRS, gastrorenal shunt; GV, gastric varices; SGV, short gastric vein; PV, portal vein; LRV, left renal vein; SMV, superior mesenteric vein; SV, splenic vein.

At the 1-month follow-up, enhanced CT examination showed that the gastroesophageal varices were completely obliterated in 24 patients (80%, 24/30), whereas partial obliteration of the gastric varices and complete obliteration of the feeding veins were achieved in the remaining 6 patients (20%, 6/30). The twenty-four patients with complete obliteration underwent CT and CT portal venography, which revealed that the gastric varices, the perforating veins in the fundus, the perifundus veins, and all the feeding veins were filled with cyanoacrylate at 1 month after the procedure (Figures 5(a) and 5(b)). From 3 to 6 months after PTAE, the amount of cyanoacrylate in the submucosal varices was lower than that observed at the beginning of follow-up, similar to the findings observed during endoscopic follow-up. However, the cyanoacrylate located in the perifundus varices and feeding veins remained in situ. One year after the procedure, the cyanoacrylate in the submucosa varices had almost disappeared, but the perifundus varices and the feeding veins remained filled with cyanoacrylate (Figure 5(c)). Portal venography showed that there was no blood flow in the obliterated varices and that all the feeding veins were sufficiently filled with cyanoacrylate 1 year after the procedure (Figure 5(c)). Moreover, the GRSs may be protected (Figure 5(c)). Newly developed ascites or aggravated ascites were observed on CT in 3 patients and were controlled by conservative therapy.

### 3.4. Rebleeding

Rebleeding was observed in 4 patients after treatment. The cumulative rebleeding rates observed at 1, 2, 3, and 5 years were 3.7%, 21.6%, 21.6%, and 21.6%, respectively (Figure 4(b)). Endoscopy was performed to identify the reason for rebleeding in these patients. In 2 patients suffering from melena (18 months and 23 months after PTAE, respectively), the rebleeding came from a peptic ulcer, was controlled by conservative therapy, and did not recur during the follow-up period. As previously mentioned, in 1 patient (1/30), rebleeding (23 months after the PTAE procedure) was caused by esophageal varices aggravation. Rebleeding due to the recurrence of gastric varices was found in only 1 patient (1/30) (5 months after the PTAE procedure). Additional endoscopic histoacryl injection was performed in this patient.

### 3.5. Changes in Hepatic Function

Overall, at 3 months after PTAE, the international normalized ratio (INR) had significantly improved (P= 0.004, paired t-test) from 1.27±0.13 (range 1.13–1.7) to 1.21±0.13 (1.01–1.5). However, the total serum protein and serum albumin levels were not significantly changed following PTAE (Table 2). A significant change (P =0.004, Wilcoxon t-test) was observed in Child-Pugh scores after the procedure (before: A/B/C = 19/8/3; after: A/B/C =22/7/1).

### 3.6. Survival Rates

During the follow-up period, 3 patients died. The first died of liver failure 5.5 months after PTAE therapy; the gastroesophageal varices did not worsen in this patient. The second patient progressed to HCC 10 months after the procedure even though he received transcatheter arterial chemoembolization (TACE), and he died of liver failure as the result of progression of HCC 28 months after the initial PTAE therapy. The third patient died of rebleeding 35 months after PTAE therapy. The mean follow-up period was 30 months (2–78 months), and the cumulative survival rates at 1, 2, 3, and 5 years were 96.3%, 96.3%, 79.9%, and 79.9%, respectively.

In addition to the patients mentioned above, two patients progressed to HCC at 17 and 38 months after the PTAE procedure. All of these patients received hepatectomy and were alive and clinically followed up at the end of the study.

## 4. Discussion

In contrast to esophageal varices, the anatomy and hemodynamic indexes of gastric varices are more complex, especially in fundal varices [22]. The optimal modality for the treatment of gastric varices has not yet been established [23]. Isolated gastric varices (IGVs) located in the gastric fundus are usually very large, frequently drain via GRSs into the left renal vein, and have an incidence of 80-85% [24]. These large GRSs may potentially increase the risk of ectopic embolism, such as pulmonary embolism, when embolism therapy is performed in the gastric varices [25, 26]. Therefore, the treatment of IGVs associated with large GRSs is challenging. Although BRTO has been recommended as the treatment of choice in patients with gastric variceal rebleeding in a recent consensus and recent guidelines [27], this procedure still has some drawbacks, such as sclerosant-related intravascular hemolysis, balloon rupture-related treatment failure, catheter-related infection, and a potential increase in esophageal varices [28]. In previous studies, we reported our successful experience in performing modified PTAE with 2-OCA in the treatment of esophageal and gastric varices, in which we achieved ideal medium- and long-term results [16–18, 20, 21, 29].

In recent years, we introduced modified PTAE performed using 2-OCA for the treatment of IGVs associated with large GRSs. During this procedure, cyanoacrylate is injected via the feeding veins into all the varices to achieve the permanent obliteration of the varices and their feeding veins. To reduce the risk of ectopic embolism, a balloon is placed at the entrance to the GRS or renal vein to temporarily occlude the GRS. In the present study, we evaluated the long-term results of this procedure in terms of rebleeding, survival, and complications.

In this study, the technical success rate was 100%, and the gastric varices were completely absent or markedly smaller in subsequent follow-ups. The total rebleeding rate was 16.7% (4/30), while rebleeding due to recurrence of gastric varices was found in only 1 (1/30, 3.4%) patient. The cumulative nonbleeding rates at 1, 2, 3, and 5 years were 96.3%, 78.4%, 78.4%, and 78.4%, respectively. In terms of patient survival, the cumulative survival rates at 1, 2, 3, and 5 years were 96.3%, 96.3%, 79.9%, and 79.9%, respectively. Progression to HCC and liver failure was the main causes of death, and only one patient died of variceal rebleeding in the present study. These results indicate that balloon-assisted percutaneous transhepatic antegrade embolization performed using 2-OCA is a feasible and effective method for the treatment of IGVs associated with GRSs.

In terms of adverse events, there were no serious procedure-related complications except for one asymptomatic pulmonary embolism caused by a small balloon, which did not block the blood flow through the shunt adequately. According to previous studies, the polymerization time of cyanoacrylate might be prolonged markedly when mixed with lipiodol [30]. Therefore, cyanoacrylate was mixed with lipiodol in purpose of tracing and prolonging the polymerization time. In addition, the mixture could diffuse more sufficiently to gastric varices, allowing for more completed embolization. However, slower-polymerizing forms of cyanoacrylates might increase the risk of ectopic embolism. Therefore, appropriate concentration of cyanoacrylate was relatively important. In previous studies, a mixture of cyanoacrylates and lipiodol with a ratio of 1:1 or 1:2 was frequently used [31]; therefore, a 50% concentration of cyanoacrylate was chosen in the current study. Unfortunately, even though the drainage vein was occluded when cyanoacrylate was injected into GVs, migration of cyanoacrylate into the pulmonary artery occurred in one case. Though no symptom was observed in this patient, it indicated that there was a possibility of ectopic embolism during or after this procedure. There were two possible reasons for the ectopic embolism: first, a small sized balloon was selected which did not adequately block the blood flow through the shunt; second, over diluted cyanoacrylates tended to float downstream before polymerization. This also reflects the importance of balloon occlusion of GRSs from one side. In the following research, the means to avoid ectopic embolism is also the focus of our work. Possible approaches were described as follows: first, increase the concentration of cyanoacrylates, such as 60-70%, to fasten the polymerization in the GVs; second, choose an adequate balloon large enough to occlude the GRS; third, the balloon should be held in place for a few minutes after the embolization, so as to ensure the completed cyanoacrylate polymerization. The stability of glue should be carefully evaluated before removing the balloon.

Although BRTO is an effective therapy to treat GV bleeding, its lengthy procedure time and the risk of balloon rupture during the indwelling time are drawbacks associated with this procedure [32]. BRTO often requires hours of postprocedure monitoring as the indwelling occlusion balloon must be kept in place long enough to achieve complete resolution of the GV [7, 13, 33–35]. Leaving the balloon for such a long time raises the potential risks of bleeding and infection and inconveniences the patient. Furthermore, balloon rupture occurs during BRTO in 8.7% of patients [36]. Once it occurs, it can cause rapid migration of the sclerosant, pulmonary embolism, or recurrent gastric variceal bleeding [36]. It can also lead to technical failure and put the patient at risk. In contrast to BRTO, in our study, we did not need to keep the balloon in place for as long an indwelling time as was required for BRTO, and the balloon could be removed immediately after PTAE. Hence, this procedure avoids the above drawbacks.

Furthermore, PTAE does not require the use of any sclerosing agents, including liquid and foam sclerosants and therefore avoids sclerosant-related complications. The most common sclerosing agent used in Japan for BRTO is ethanolamine oleate (EO) [6, 7, 11, 33, 37–40]. However, EO causes hemolysis, which may result in renal tubular disturbances and acute renal failure. Haptoglobin has been used as the antidote to this condition [41]; however, it cannot be easily obtained outside of Japan. Other reported complications associated with the use of EO include pulmonary edema, cardiogenic shock, disseminated intravascular coagulation, and anaphylactic reactions [41–44]. To overcome these potential shortcomings of EO, some researchers have tried to use foam sclerosants in BRTO. However, some complications are also associated with foam sclerosants, such as pulmonary edema and partial portal vein thrombosis [45–47]. In our study, no sclerosing agents were required; hence, no complications associated with sclerosing agents were observed. This is similar to plug-assisted retrograde transvenous obliteration (PA-RTO) and coil-assisted retrograde transvenous obliteration (CA-RTO), which were developed in recent years and to occlude GRSs and GVs using plugs or coils and gelatin sponges instead of sclerosing agents [28, 48, 49].

The most essential difference between PTAE and BRTO is that, in the PTAE procedure, the gastric varies are embolized antegradely through the efferent veins, and the GRS is not embolized and might still be a potential shunt for portal vein blood flow. One of the most important reported complications associated with BRTO is aggravated esophageal varices [50]. The probable reason is that, in the BRTO procedure, the gastric varices were embolized through the outflow vein, which means that the spontaneous portal-systemic shunt (mainly the GRS) was embolized as well as the gastric varices. However, the inflow veins may not be completely embolized. Once the GRS is obstructed, the flow and pressure in the portal vein increase [44]. As a result, new collateral veins to the esophageal varices develop, worsening the esophageal varices [44]. In contrast, PTAE not only embolizes all gastric varices but it also embolizes all of the inflow veins (i.e., the left gastric vein, short gastric vein, and/or posterior gastric vein), which are the potential inflow vessels of esophageal varies. Moreover, the spontaneous portal-systemic shunt (mainly the GRS) may be reserved (Figure 5(c)). Therefore, in the present study, although portal vein pressure (PVP) increased from 24.3 (8-47.8 mmHg) to 29.1 (10-48.6 mmHg), the rate at which esophageal varices worsened was only 13%, and this was lower than the rate reported for BRTO (up to 63%, with 11–24% of cases showing subsequent variceal bleeding) [51, 52].

The worsening of ascites and/or hydrothorax has also been reported to be another problem after BRTO. In the present study, in a 3-month follow-up CT or B-US, ascites had newly developed in three of 30 patients (10%) who did not have ascites before PTAE, and ascites progressed to a larger amount in 1 of 30 patients (3.4%) who had ascites before PTAE. All of these patients were controlled by medical therapy, and none of them developed refractory ascites. These results appeared to be equal to the variable results (0–44%) [11, 46, 53, 54] reported after BRTO, although these evaluations have not been standardized.

Similar to BRTO, one potential benefit of PTAE is that it improves hepatic function. In BRTO [6, 11, 55], improvement in liver function was shown to be dependent on an increase in portal hepatic blood flow. A study by Kumamoto et al. [55] provided evidence that BRTO might have a role in preserving liver function. They reported transient improvement in hepatic function, with results returning to baseline levels by 3 years, similar to results reported in patients without gastrosystemic shunts with stable hepatic function. These findings suggest that occlusion of the GRS may protect the liver from portosystemic shunt syndrome and have a protective long-term role in preserving hepatic function. In the present study, improvement in Child–Pugh scores was observed in 11 of 30 patients (36.7%) within 3 months after PTAE, and INR was also significantly improved. This suggests that hepatic function may also be improved after PTAE in the short-term. However, there was no long-term follow-up of hepatic function.

The PTAE procedure has some limitations, including the fact that a transhepatic approach is needed. Hepatic puncture tract bleeding is a potential complication of this technique. To overcome this shortcoming, in every patient, the puncture tract was embolized with a coil, and a gelatin sponge was applied if necessary. No puncture tract bleeding was observed. The limitations of our preliminary study include its retrospective design, the limited number of patients, and the absence of long-term follow-up. Prospective, randomized, comparative trials with large numbers of patients, and longer follow-up periods are necessary to better evaluate the benefits of PTAE.

In conclusion, our preliminary results suggest that PTAE is technically feasible and safe. This procedure seems to be clinically effective for the treatment of IGV with large GRSs. Therefore, PTAE is useful as a therapeutic strategy in the treatment of IGV associated with large GRSs, at least as a useful complement to BRTO.

## Figures and Tables

**Table 1:** Patient characteristics.

Characteristics	Patients
Age (years)	49.5 (26-81)
Sex (male/female)	16/14
Etiology of cirrhosis	
HBV/HCV	17/1
Alcohol/autoimmunity/unknown	6/3/3
Child-Pugh classification	
A/B/C	19/8/3
Location of gastric varices	
IGV1	30
Form of gastric varices	
F2	11
F3	19
Presence of red spots	14
Encephalopathy	2
Gastrorenal shunt	30
History of variceal bleeding	30
Follow-up period (mean)	30 months (2-80)

**Table 2:** Changes in liver function parameters 3 months after PTAE.

Parameters	pre-PTAE	3 Months After PTAE	95% Confidence Interval		P Value	t Value
Serum total bilirubin level (*μ*mol/L)	27.59±23.25	21.84±10.70	-2.45	13.95	0.162*∗*	1.435
Serum albumin level (g/L)	34.07±5.72	35.17±5.18	-2.77	0.56	0.184*∗*	-1.36
INR	1.27±0.13	1.21±0.13	0.02	0.1	0.004*∗*	3.174
Child-Pugh classification						
A/B/C	19/8/3	22/7/1			0.009*∗*	

*∗*Data collected before and after PTAE were compared to analyze hepatic function data using the paired t-test.

*∗∗*Data collected before and after PTAE for the Child-Pugh classification were analyzed using the Wilcoxon t-test.

INR, international normalized ratio.

## Data Availability

The data used to support the findings of this study are available from the corresponding author upon request.
